# ShinyTHOR app: Shiny-built tumor high-throughput omics-based roadmap

**DOI:** 10.1093/bioadv/vbaf061

**Published:** 2025-03-21

**Authors:** Eduardo Navarrete-Bencomo, Anthony Vladimir Campos Segura, Orlando R Sevillano, Ana Mayanga, José Luis Buleje Sono, César Alexander Ortiz Rojas, Alexis Germán Murillo Carrasco

**Affiliations:** Unidad Profesional Interdisciplinaria de Biotecnología del Instituto Politécnico Nacional, Av. Luis Enrique Erro S/N, Zacatenco, Alcaldía Gustavo A. Madero, Ciudad de México, CP 07738, México; Centro de Ciencias Genómicas, UNAM, Av. Universidad S/N, Universidad Autónoma del Estado de Morelos, Cuernavaca, Morelos 62210, México; Clinical and Functional Genomics Group, International Center of Research CIPE, A.C.Camargo Cancer Center, Sao Paulo 01509-010, Brazil; Research Group in Biochemistry and Synthetic Biology (GIBBS-UNFV), Lima, Lima 15007, Peru; Immunology and Cancer Research Group (IMMUCA), OMICS, Lima, Lima 15001, Peru; Laboratório de Soroepidemiologia e Imunobiologia, Instituto de Medicina Tropical, Faculdade de Medicina, Universidade de São, São Paulo 05403-000, Brazil; Cancer and Stem Cells Research Group, Universidad Científica del Sur, Lima 15067, Peru; Centro de Investigación en Genética y Biología Molecular, Facultad de Medicina Humana, Universidad de San Martín de Porres, Lima, Lima 15011, Peru; Immunology and Cancer Research Group (IMMUCA), OMICS, Lima, Lima 15001, Peru; Laboratório de Investigação Médica (LIM) 31, Hospital das Clínicas HCFMUSP, Faculdade de Medicina, Universidade de São Paulo, São Paulo, SP 01246-000, Brazil; Immunology and Cancer Research Group (IMMUCA), OMICS, Lima, Lima 15001, Peru

## Abstract

**Motivation:**

The Cancer Cell Line Encyclopedia (CCLE), launched in 2008, systematically organizes multi-omic and pharmacological data from over 1000 cancer cell lines with molecular dependency maps accessible through the DepMap tool. However, DepMap lacks tools for systematic comparison of mRNAs, miRNAs, proteins, and metabolites, as well as their links to drug responses and gene signatures. Extracting this data externally requires bioinformatics expertise, limiting access for wet-lab researchers.

**Results:**

We developed ShinyTHOR, a web app for intuitive access to multi-omic (transcriptomic, metabolomic, methylomic, proteomic, and miRNomic) and drug-related data. It integrates datasets from CCLE, miRTarBase, circInteractome, and the Genomics of Drug Sensitivity in Cancer. ShinyTHOR includes six modules: (1) identifying top/bottom ten cell lines by marker expression or drug IC50, (2) single-sample Gene Set Enrichment Analysis (ssGSEA), (3) multi-analyte expression evaluation, (4) miRNA-target interactions across cancer types, (5) miRNA impact on mRNA translation via protein levels, and (6) circRNA/miRNA modulation. To validate its utility, we applied ShinyTHOR to a gastric cancer prognosis study (GES7).

**Availability and implementation:**

ShinyTHOR is freely accessible for non-commercial use at https://alexismurillo.shinyapps.io/ShinyThor, with source code available at https://github.com/Murillo22/ShinyThor.

## 1 Introduction

Cell lines have been invaluable tools in cancer research, providing reproducible systems to test hypotheses, evaluate anticancer agents, and study genetic and epigenetic alterations in controlled environments (Gillet *et al.* 2013, Klijn *et al.* 2015). Recent advances have made omics and drug screening data from cell lines publicly available through databases like the Cancer Cell Line Encyclopedia (CCLE), part of the Cancer Dependency map (DepMap) project (Barretina *et al.* 2012). These resourcers promote transparency, reproductibility, and the reuse of data across studies, enabling novel insights beyond their original scope (Ghandi *et al.* 2019).

The CCLE, launched in 2008, systematically organizes multi-omic and pharmacological data from over 1000 cancer cell lines (Barretina *et al.* 2012). Its goals include genetic and cellular features, and translating genomic findings into cancer patient stratification (Ghandi *et al.* 2019). While DepMap harmonizes and provides access to these datasets, it lacks native tools for systematic comparison of mRNAs, miRNAs, proteins, and metabolites, as well as their associations with drug responses and gene signatures. Extracting and analyzing this data externally often requires significant bioinformatics expertise, posing challenges for wet-lab researchers (Zheng *et al.* 2020).

To address this, we developed *ShinyTHOR* (Tumor High-throughput Omics-based Roadmap on Shiny), a free, open-access web app designed for wet-lab researchers. *ShinyTHOR* simplifies data integration and analysis by enabling users to evaluate associations between omic analytes, drug responses, and gene signatures across selected cancer cell lines. Built using R and HTML through ShinyApps, the app combines CCLE data visualization with an intuitive interface, streamlining experimental planning in cancer research.

## 2 Methods

### 2.1 Implementation

This is a summarized overview of our software implementation. For detailed information and the user guide, please refer to Supplementary Material S1.

#### 2.1.1 Analyte expression component

In this component, we organize the omics data according to these five modules: “Gene expression,” “miRNA expression,” “Protein expression,” “Metabolite expression,” “Methylation levels,” and “Drug IC50.” The “Methylation levels” module allows users to evaluate methylation levels in promoter regions using the following format: *Gene_Chromosome_StartPosition_EndPosition*. Once the target is selected, it is possible to delimit the list of cell lines that will be evaluated through four filters; the first three are associated with the affected organ, histology, or type of pathology. The fourth filter is manual, therefore, it is possible to review the cell lines that will be analyzed. Finally, by the “top/bottom” option, you can choose the top or bottom ten cell lines for this target. As a result of this component, users will see a barplot showing the levels of the top/bottom 10 cell lines for the target selected in the “Analyte profile” tab (based on the “ggplot2” package). Then, a high-resolution image (600 dots per inch, 600 dpi) can be downloaded.

#### 2.1.2 Scoring gene signatures of biological pathways

Single-sample gene set enrichment analysis (ssGSEA) was utilized to calculate enrichment scores based on the coordinated differential regulation of gene sets, similar to the classic gene set enrichment analysis (GSEA) algorithm. However, unlike the traditional GSEA, which computes enrichment scores for groups of samples, ssGSEA calculates these scores for each sample.

#### 2.1.3 Multiple analyte component

In this module, it is possible to evaluate the expression levels of several targets simultaneously as long as they belong to the same module (among the four available: Gene, miRNA, Protein, and Metabolite modules). The system starts by indicating the module of interest. Then, the different targets must be entered, one for each line. After filtering the cell lines to be displayed, the Heatmap tab will show a heatmap with the scaled expression of the participating targets. This heatmap is produced with the “superheat” package and can be downloaded in 600-dpi resolution.

#### 2.1.4 miRNA-gene component

Here, inverse correlations can be found once miRNAs negatively suppress the transcription of relative genes. In this component, we pre-filter the relationships between genes and miRNAs based on the interactions described by miRTarBase to evaluate, at a cell line level, whose fulfill repression features. In the first filtering point, users can choose between start choosing genes or miRNAs. Depending on that choice, a list of genes or miRNAs with available information will appear. Once the first region of interest is selected, the list of potential targets (second region of interest) will be updated (according to miRTarBase). Then, the three additional input information will filter the list of cell lines with available information.

#### 2.1.5 miRNA–protein component

In this module, users can find correlations about the effect of miRNA expression against the protein levels. microRNAs affect protein levels by binding to the mRNA of a target gene, which can lead to either the degradation of the mRNA or inhibition of its translation, ultimately resulting in a decrease in the production of the corresponding protein; essentially, miRNA act as negative regulators of gene expression at the post-transcriptional level, lowering protein levels without altering the DNA sequence itself.

#### 2.1.6 Modulation tools component

Finally, this component compiles information on circRNAs and miRNAs that potentially repress the expression of a selected gene and can be used to synthesize new sequences to run a gene-silencing experiment. In this component, it is possible to select a gene from the list, and the system will display the list of miRNAs that potentially block the gene (from the miRTarBase) and a link to the corresponding circInteractome portal, where different circRNA options can be found.

## 3 Conclusions

The ShinyTHOR tool could significantly contribute by providing previous results of genetic alterations and cell line models. In this article, we take the previous study of GES7 (Velásquez Sotomayor *et al.* 2023) in stomach carcinoma cell lines (Supplementary Material S1) as an example to show the tool’s usefulness. However, ShinyTHOR could also complement the evaluation of many other *in silico* studies in different types of tumors, such as breast, esophagus, intestine, lung, skin, etc.

## 4 Perspectives

Future versions of the ShinyTHOR app aim to expand its functionality by incorporating additional datasets and allowing users to define their cell lines.

## Supplementary Material

vbaf061_Supplementary_Data

## Data Availability

Data including mRNA, miRNA, protein, and metabolites, were retrieved from the CCLE database (https://depmap.org/portal/data_page). We also complemented these observations with IC50 concentrations for selected drugs from the Genomics of Drug Sensitivity in Cancer portal (https://www.cancerrxgene.org/downloads/bulk_download). The ShinyThor web app is freely available to non-commercial users at https://alexismurillo.shinyapps.io/ShinyThor, and the source code can be accessed at https://github.com/Murillo22/ShinyThor.

## References

[vbaf061-B1] Barretina J , Caponigro G, Stransky N et al The Cancer Cell Line Encyclopedia enables predictive modelling of anticancer drug sensitivity. Nature 2012;483:603–7. 10.1038/nature11003 22460905 PMC3320027

[vbaf061-B2] Ghandi M , Huang FW, Jané-Valbuena J et al Next-generation characterization of the cancer cell line encyclopedia. Nature 2019;569:503–8. 10.1038/s41586-019-1186-3 31068700 PMC6697103

[vbaf061-B3] Gillet JP , Varma S, Gottesman MM. The clinical relevance of cancer cell lines. J Natl Cancer Inst 2013;105:452–8. 10.1093/JNCI/DJT007 23434901 PMC3691946

[vbaf061-B4] Klijn C , Durinck S, Stawiski EW et al A comprehensive transcriptional portrait of human cancer cell lines. Nat Biotechnol 2015;33:306–12. 10.1038/NBT.3080 25485619

[vbaf061-B5] Velásquez Sotomayor MB , Campos Segura AV, Asurza Montalva RJ et al Establishment of a 7-gene expression panel to improve the prognosis classification of gastric cancer patients. Front Genet 2023;14:1206609. 10.3389/FGENE.2023.1206609 37772256 PMC10522918

[vbaf061-B6] Zheng H , Zhang G, Zhang L et al Comprehensive review of web servers and bioinformatics tools for cancer prognosis analysis. Front Oncol 2020;10:68. 10.3389/FONC.2020.00068 32117725 PMC7013087

