# Supplementary material for: ShinyTHOR app: Shiny-built tumor high-throughput omics-based roadmap
Source: Bioinform Adv. 2025 Mar 21;5(1):vbaf061. doi: 10.1093/bioadv/vbaf061 (PMC12085240; doi:10.1093/bioadv/vbaf061)
Supplement: vbaf061_Supplementary_Data [file vbaf061_supplementary_data.docx]

# Supplement to “ShinyTHOR app: Shiny-built Tumor High-throughput Omics-based Roadmap”

**Authors:** Eduardo Navarrete Bencomo, Anthony Vladimir Campos Segura, Orlando R. Sevillano, Ana Mayanga, José Luis Buleje Sono, César Ortiz & Alexis Germán Murillo Carrasco

# USER GUIDE

# Implementation

## Data sources

Data, including mRNA, miRNA, protein, methylated regions, and metabolites, were retrieved from the CCLE database [1]. We also complemented these observations with IC50 concentrations for selected drugs from the Genomics of Drug Sensitivity in Cancer portal [2]. Here, we briefly describe the origin of each dataset.

### *RNA expression profiling*

The RNA profiling data in the DepMap project arises from sequencing and analysis of 1,019 cell lines using Illumina’s TruSeq RNA preparation protocol, selected polyadenylated mRNA using oligo-dT beads, and cDNA synthesis after heat fragmentation [1]. In the app, we used log2(TPM+1) as the unit to measure the RNA expression.

### *miRNA expression profiling*

Data concerning miRNA profiling was obtained using a high-performance RNA validation NanoString platform, which enables reliable and reproducible evaluation of levels for multiple regions​ [3].

The CCLE miRNA profiling data were obtained as described by Ghandi et al. [4]. In summary, they processed 734 miRNAs in 954 cell lines using the NanoString Platform following the manufacturer’s instructions. The purified probes were counted using nCounter (NanoString Technologies). Relative Units were used to measure the levels of miRNA.

### *Reverse Phase Protein Analysis (RPPA)*

Protein abundance data comes from Reverse Phase Protein Analysis (RPPA), an antibody-based technique. An RPPA consists of immobilizing thousands of different cell lysates on a nitrocellulose-coated slide, followed by incubations with protein-specific antibody detection. Quantification of the protein expression is typically performed using a standard curve generated with an antibody detection probe [5]. Detection and quantification were performed following an MD Anderson Cancer Center pipeline. After the Reverse Protein Analysis measures the Signal Intensity (fluorescence intensity), this measurement is processed to estimate the relative protein expression level and normalized by the median value [5]. RPPA was available for 899 cell lines.

### *Metabolite abundance profiling*

For metabolite abundance measurements, 928 cancer cell lines from 20 major cancer types were cultured to obtain metabolomic profiles of 124 polar species and 101 lipid species [6,7]. Metabolites were analyzed by hydrophilic interaction chromatography (HILIC) and reverse-phase chromatography. Ample measurements were performed in four batches using pooled lysates as references to ensure consistent data quality. The abundance of each metabolite is measured in log10(level) [6].

### *Methylated regions profiling*

The CCLE DNA methylation profile used Illumina Infinium arrays (450K/850K), covering CpG sites across promoters, gene bodies, and enhancers [1,6]. The data, represented as relative β-values (0–1), reveal key epigenetic patterns, such as global hypomethylation in intergenic regions and promoter hypermethylation of tumor suppressor genes. Cancer type-specific methylation signatures correlate with gene expression, influencing oncogene activation and tumor suppressor silencing. The dataset enables cross-cancer comparisons, biomarker discovery, and epigenetic drug response studies, supporting cancer classification and therapeutic research.

### *Drug Sensitivity data*

IC50 data were retrieved from the Genomics of Drug Sensitivity in Cancer (GDSC) portal (<https://www.cancerrxgene.org/>) [2]. Two datasets were considered: GDSC1, which contains IC50 data for 970 cell lines against 403 compounds by Resazurin or Syto06 assay; and GDSC2, which contains data for 969 cell lines against 297 compounds by CellTitreGlo assay. For plotting, we consider the concentration of each drug in µM.

### *miRNA-gene Interactions*

MicroRNAs (miRNAs) are noncoding RNAs with around 18-26 nucleotides in length and are found to be part of regulatory systems in plants, fungi, animals, and protozoa [8]. Due to their role in different cellular processes, miRNAs are related to many types of diseases, and clinical trials based on miRNAs are encouraging in this alternative treatment of cancer and viral infections [8,9]. mirTarBase is a database where we can find curated information about miRNA-target interactions (MTIs) and miRNA expression profiles [10]. As of the review of building this app, mirTarBase has accumulated over 2,200,000 verified MTIs from the manual curation of 19,389 articles and CLIP-seq data. mirTarBase has integrated some types of data, such as gene- and miRNA-specific SNPs and DRVs, miRNA-disease association database, regulation of miRNAs, miRNA expression, and editing events in miRNAs; this information is in specific databases integrated in mirTarBase [10].

### *circInteractome*

circInteractome [11] allows researchers to map binding sites for RBP (RNA Binding Proteins) and miRNA in human circRNA. circInteractome searches in public circRNA, miRNA, and RBP databases to provide bioinformatic analyses of binding sites in junction-flanking sequences. circInteractome allows the user to identify potential circRNAs with possible functions as RBP sponges, design junction-spanning primers for specific detection of circRNAs of interest, design small interfering RNAs (siRNAs) for circRNA silencing, and identify potential internal ribosome entry sites.

## Analyte Expression component

In this component, we offer the possibility of selecting cell lines that express the highest or lowest levels of a certain target. We organize the omics data according to these six modules: “Gene expression”, “miRNA expression”, “Protein expression”, “Metabolite expression”, “Methylation levels”, and “Drug IC50”. Then, there is an option to choose the corresponding module. Once the module is selected, the option to choose the specific target (one of 19,193 genes, 654 miRNAs, 144 proteins, 225 metabolites, 20192 methylation levels, or 542 drugs available in the system) is enabled.

Once the target is selected, it is possible to delimit the list of cell lines that will be evaluated through 4 filters, the first 3 are associated with the affected organ, histology, or type of pathology. The fourth filter is manual, it is possible to review cell lines that will be analyzed. Finally, by the “top/bottom” option, you can choose the top or bottom ten cell lines for this target.

As a result of this component, users will see a barplot showing the levels of the top/bottom 10 cell lines for the target selected in the "Analyte profile" tab (based on the "ggplot2" package). Then, a high-resolution image (600 dots per inch, 600 dpi) can be downloaded. In addition, the "Analyte Data" tab shows all the cell lines filtered in the input and their respective expression levels for the selected analyte. Users can freely download this data to have a backup or produce additional analyses. Finally, the "Anatomic expression" tab is a preliminary approach to show the average target expression across all organs mapped in CCLE, depending on the gender information (male or female) of the patient that gives the sample for developing each cell line. This image is generated based on the "gganatogram" R package. This anatomic-level function is on a beta test, we expect to collect users' opinions and convert this tab into a separate module.

## Scoring gene signatures of biological pathways

Single-sample gene set enrichment analysis (ssGSEA) was utilized to calculate enrichment scores based on the coordinated differential regulation of gene sets, similar to the classic GSEA algorithm. However, unlike the traditional GSEA that computes enrichment scores for groups of samples, ssGSEA calculates these scores for each sample. This approach allows for more granular analysis of gene expression data, capturing the unique biological pathway activities of individual samples. The ssGSEA was performed using R version 4.4.1, employing a script by Pranali (https://rpubs.com/pranali018/SSGSEA). This methodology follows the procedure described by Barbie et al. [12], ensuring consistency with established protocols. In detail, ssGSEA involves: Gene Set Ranking, where each gene in the dataset is ranked based on its expression level within each sample; Enrichment Score Calculation, where for each predefined gene set, an enrichment score is computed by evaluating the overrepresentation of the genes in the ranked list of each sample; and normalization, where the enrichment scores are normalized to account for differences in gene set sizes and other potential biases. For ShinyTHOR, 10,461 gene ontology (GO) gene sets were considered, which contain GO biological process (BP), GO cellular components (CC), and GO molecular function (MF) [13].

## Multiple Analyte component

In this module, it is possible to evaluate the expression levels of several targets simultaneously as long as they belong to the same module (among the four available: Gene, miRNA, Protein, and Metabolite modules). Here, it is also possible to filter the cell lines to be shown according to the affected organ, type of histology, and type of pathology.

The system starts by indicating the module of interest. Then, the different targets must be entered, one for each line (in the “Available analytes” tab, it is possible to review the list of targets available for each module. After filtering the cell lines to be displayed, the Heatmap tab will show a heatmap with the scaled expression of the participating targets. This heatmap is produced with the “superheat” package and can be downloaded in 600-dpi resolution.

## miRNA-gene component

To the best of our knowledge, gene expression levels tend to be affected by the production of specific miRNAs. Here, inverse correlations can be found once miRNAs negatively suppress the transcription of relative genes. This approach is proposed to be an effective way to identify miRNA target pairs [14,15]. For instance, Ferraz et al. (2013) identified that down-expressed miR-31 promotes tumorigenesis and dedifferentiation of proliferation mechanisms by increasing cyclin D1 expression and modulating cell cycle progression [15]. Thus, an inverse correlation relationship is found in the expression of miR-31 and cyclin D1. Naturally, this repression depends on the notable expression of the miRNA or gene.

In this component, we pre-filter the relationships between genes and miRNAs based on the interactions described by miRTarBase [10] to evaluate, at a cell line level, which genes fulfill repression features. This component has six input fields to be filled. In the first filtering point, users can choose between starting choosing genes or miRNAs. Depending on that choice, a list of genes or miRNAs with available information will appear. Once the first region of interest is chosen, the list of potential targets (the second region of interest) will be updated (according to miRTarBase). Then, the three additional input information will filter the list of cell lines with available information.

After choosing the selected gene and the corresponding miRNA, the system will display their levels in the pre-filtered list of cell lines using a dynamic graph (produced using the “plotly” package). The “miRNA-gene profile” will show a scatterplot where each point represents a cell line. The y-axis will show the levels of the selected gene, whereas the x-axis will show the miRNA levels for the user’s choice. It is then possible to mouse over these spots to obtain cell line information and independent levels of the participating miRNA and gene. Additionally, the “miRNA-gene Data” tab displays the tabulated data.

## miRNA-protein component

According to the data in the CCLE through RPPA data, we adapted a Module that the users can asses the effect of specific miRNA against one of the 144 possible proteins to select in ShinyTHOR version 2.2. Users can visualize a similar plot as in the miRNA-gene component, a scatterplot in the miRNA profile tab, where each point represents a cell line. On the y-axis, we can find the protein level in relative units, and on the x-axis, the miRNA of interest in the same units as the protein level.

The module needs five inputs: the miRNA of interest, the protein of interest, followed by the choice of a specific group of cell lines, the histologic and pathologic group. The miRNA-protein profile tab shows the scatterplot mentioned above, and the miRNA-protein Data tab shows the information used to create the plot in table form.

## Modulation tools component

Finally, this component compiles information on circRNAs and miRNAs that potentially repress the expression of a selected gene and can be used to synthesize new sequences to run a gene-silencing experiment. In this component, it is possible to select a gene from the list, and the system will display the list of miRNAs that potentially block the gene (from the miRTarBase) and a link to the corresponding circInteractome portal, where different circRNA options can be found. After assessing these functions between users, is planned to be added to download the sequence of the valid transcript of the selected gene. Therefore, users could use this FASTA sequence to evaluate siRNAs in the siDirect tool [16].

# Results and user guide

## Frontpage

ShinyThor can be freely accessed by this link: <https://alexismurillo.shinyapps.io/ShinyThor/>. After loading all main functions, the app will display a front page with database features (**Figure 1**). This page includes the current version of the app (2.0), the release date (25-06-2024), as well as the number of available analytes and cell lines.

## Example case

Gastric cancer ranks fifth in incidence and fourth in mortality worldwide [17]. The high mortality rate of patients with gastric cancer requires new biomarkers that allow early and efficient diagnosis to provide the patient with adequate treatment [18]. A previous study identified a panel of 7 genes (*CCDC91, DYNC1I1, FAM83D, LBH, SLITRK5, WTIP,* and *NAP1L3*) that are important in the development of gastric cancer [18].

In this hypothetical case, researchers aim to evaluate the performance of GES7 genes in locally available gastric cancer cell lines (KATOIII and AGS). Then, an analysis seeking to determine the expression levels for GES7 genes in KATO III and AGS can be performed through ShinyTHOR.

## Evaluating the expression of the *CCDC91* gene

In this module, it is possible to select relevant options such as the proper module (Gene Expression, miRNA Expression, Protein expression, Metabolite Expression, or Drug IC50), the region of interest, and the specific group, histology, or pathologic group of cell lines. In **Figure 2**, an example is shown of graphing the expression levels of the *CCDC91* gene in stomach carcinoma cell lines. In addition, a table with all the information related to the query can be visualized, where you can find a summary of a hit of the gene of interest and its expression level in different cell lines, primary sites, histology, and pathology (**Figure 3**).

## Evaluating relevant Gene Ontology pathways in the KATO III cell line

In the ssGSEA module, users can perform a single-sample Gene Set Enrichment Analysis (ssGSEA) using any available cell lines. In this hypothetical case, we performed an ssGSEA for the KATO III cell line with the parameters shown in **Figure 4**. There, it is possible to visualize the pathways positively or negatively enriched according to the calculated score. For instance, the first sets *"REGULATION OF CALCIUM ION EXPORT ACROSS PLASMA MEMBRANE"* and *"PEROXYREDOXIN ACTIVITY"* shows positive enrichment in this cell line, whereas *"OLFACTORY RECEPTOR ACTIVITY"*, *"ODORANT BINDING"* and *"CCR1 CHEMOKINE RECEPTOR BINDING"* are subregulated pathways according to their ssGSEA score (**Figure 4**).

## Evaluating the expression of GES7 genes

Then, researchers aimed to evaluate the expression profiles of a gene set (GES7). This is possible through the second module named “Multiple Analytes”. The first step is to select a module (Gene expression). In the second step, researchers typed some genes belonging to the GES7 panel (*CCDC91, DYNC1I1, FAM83D, LBH, SLITRK5, WTIP*, and *NAP1L3*). It is possible to click on the “Check Available Analytes” option to open a pop-up window showing the available regions for the selected module. Next, they used the following filter to choose cell lines from stomach carcinoma. The result is shown in **Figure 5**. The output shows a heatmap of GES7 genes and their respective expression levels in CCLE stomach carcinoma cell lines.

## Evaluating correlation profiles between *FAM83D* and hsa-miR-129-5p

After the last result, researchers note that *FAM83D* and *CCDC91* are notably expressed in the heatmap from **Figure 5**. Then, they wanted to evaluate these two genes due to their expression levels in the AGS and KATOIII cell lines. The researchers want to know if a difference exists between the pre-selected miRNAs for every region. They searched the correlation profile between *FAM83D* and hsa-miR-129-5p in CCLE stomach carcinoma by comparing the cell profiles of metastatic cells against primary lines (**Figures 6 and 7**). Although the results do not show a significant correlation (p-value<0.05), researchers observe a negative tendency in primary CCLE cell lines (p. e. AGS), in contrast to metastatic cell lines (p.e. KATOIII). This comparison is also available for miRNA-protein pairs (**Figures 8 and 9**).

## Looking for putative silencing regions for *FAM83D*

Finally, researchers got interested in the silencing tools for these genes. Notably, they expected to find hsa-miR-129-5p as a potential region targeting *FAM83D*. However, they want to know if other RNA-based regions could suppress this gene. This consultation is possible in the last Module called “Modulation tools” of ShinyTHOR. The only necessary step is to select the gene of interest, and a panel with the silencing tools available for the gene will be deployed, as shown in **Figure 10**. Note additional circRNA-based silencing options for this query as a direct link to the cirCinteractome database.

- 1. **Evaluating correlation profiles between P04637 and hsa-miR-129-5p**

In addition, researchers could be interested in whether miRNA (hsa-miR-129-5p) affects the protein levels of P40637 (Cellular tumor antigen p53) through miRNA expression. This analysis can be performed in the miRNA-protein Module of ShinyTHOR. The user only needs to select the miRNA and the protein of interest. After that, researchers could choose a specific cell line, histologic, and pathologic groups, such as stomach, carcinoma, and metastasis, as Figure 8 shows, respectively. Here, it is possible to inspect the plot and the results do not show a significant correlation (p-value<0.05) and a negative tendency with R equals -0.16.

# References

[1] Barretina J, Caponigro G, Stransky N, Venkatesan K, Margolin AA, Kim S, et al. The Cancer Cell Line Encyclopedia enables predictive modelling of anticancer drug sensitivity. Nature 2012 483:7391 2012;483:603–7. <https://doi.org/10.1038/nature11003>.

[2] Yang W, Soares J, Greninger P, Edelman EJ, Lightfoot H, Forbes S, et al. Genomics of Drug Sensitivity in Cancer (GDSC): a resource for therapeutic biomarker discovery in cancer cells. Nucleic Acids Res 2013;41. <https://doi.org/10.1093/NAR/GKS1111>.

[3] Goytain A, Ng T. NanoString nCounter Technology: High-Throughput RNA Validation. Methods in Molecular Biology 2020;2079:125–39. <https://doi.org/10.1007/978-1-4939-9904-0_10>.

[4] Ghandi M, Huang FW, Jané-Valbuena J, Kryukov G V., Lo CC, McDonald ER, et al. Next-generation characterization of the Cancer Cell Line Encyclopedia. Nature 2019 569:7757 2019;569:503–8. <https://doi.org/10.1038/s41586-019-1186-3>.

[5] Bioinformatics Pipeline: Protein Expression - GDC Docs n.d. https://docs.gdc.cancer.gov/Data/Bioinformatics_Pipelines/RPPA_intro/ (accessed July 7, 2024).

[6] Li H, Ning S, Ghandi M, Kryukov G V., Gopal S, Deik A, et al. The landscape of cancer cell line metabolism. Nature Medicine 2019 25:5 2019;25:850–60. <https://doi.org/10.1038/s41591-019-0404-8>.

[7] Nusinow DP, Szpyt J, Ghandi M, Rose CM, McDonald ER, Kalocsay M, et al. Quantitative Proteomics of the Cancer Cell Line Encyclopedia. Cell 2020;180:387-402.e16. <https://doi.org/10.1016/J.CELL.2019.12.023/ATTACHMENT/564C2B29-7019-423B-A0BB-487D969188B7/MMC7.XLSX>.

[8] Bartel DP. MicroRNAs: Genomics, Biogenesis, Mechanism, and Function. Cell 2004;116:281–97. <https://doi.org/10.1016/S0092-8674(04)00045-5>.

[9] Saliminejad K, Khorram Khorshid HR, Soleymani Fard S, Ghaffari SH. An overview of microRNAs: Biology, functions, therapeutics, and analysis methods. J Cell Physiol 2019;234:5451–65. <https://doi.org/10.1002/JCP.27486>.

[10] Huang HY, Lin YCD, Cui S, Huang Y, Tang Y, Xu J, et al. miRTarBase update 2022: an informative resource for experimentally validated miRNA–target interactions. Nucleic Acids Res 2022;50:D222–30. https://doi.org/10.1093/NAR/GKAB1079.

[11] Dudekula DB, Panda AC, Grammatikakis I, De S, Abdelmohsen K, Gorospe M. CircInteractome: A web tool for exploring circular RNAs and their interacting proteins and microRNAs. RNA Biol 2016;13:34–42. <https://doi.org/10.1080/15476286.2015.1128065>.

[12] Barbie DA, Tamayo P, Boehm JS, Kim SY, Moody SE, Dunn IF, et al. Systematic RNA interference reveals that oncogenic KRAS-driven cancers require TBK1. Nature 2009 462:7269 2009;462:108–12. <https://doi.org/10.1038/nature08460>.

[13] Subramanian A, Tamayo P, Mootha VK, Mukherjee S, Ebert BL, Gillette MA, et al. Gene set enrichment analysis: A knowledge-based approach for interpreting genome-wide expression profiles. Proc Natl Acad Sci U S A 2005;102:15545–50. https://doi.org/10.1073/PNAS.0506580102/SUPPL_FILE/06580FIG7.JPG.

[14] Li X, Yu X, He Y, Meng Y, Liang J, Huang L, et al. Integrated Analysis of MicroRNA (miRNA) and mRNA Profiles Reveals Reduced Correlation between MicroRNA and Target Gene in Cancer. Biomed Res Int 2018;2018:1972606. <https://doi.org/10.1155/2018/1972606>.

[15] Ferraz C, Lorenz S, Wojtas B, Bornstein SR, Paschke R, Eszlinger M. Inverse Correlation of miRNA and Cell Cycle-Associated Genes Suggests Influence of miRNA on Benign Thyroid Nodule Tumorigenesis. J Clin Endocrinol Metab 2013;98:E8–16. <https://doi.org/10.1210/JC.2012-2564>.

[16] Naito Y, Yoshimura J, Morishita S, Ui-Tei K. SiDirect 2.0: Updated software for designing functional siRNA with reduced seed-dependent off-target effect. BMC Bioinformatics 2009;10:1–8. <https://doi.org/10.1186/1471-2105-10-392/FIGURES/4>.

[17] Sung H, Ferlay J, Siegel RL, Laversanne M, Soerjomataram I, Jemal A, et al. Global Cancer Statistics 2020: GLOBOCAN Estimates of Incidence and Mortality Worldwide for 36 Cancers in 185 Countries. CA Cancer J Clin 2021;71:209–49. <https://doi.org/10.3322/CAAC.21660>.

[18] Velásquez Sotomayor MB, Campos Segura AV, Asurza Montalva RJ, Marín-Sánchez O, Murillo Carrasco AG, Ortiz Rojas CA. Establishment of a 7-gene expression panel to improve the prognosis classification of gastric cancer patients. Front Genet 2023;14. https://doi.org/10.3389/FGENE.2023.1206609/FULL.


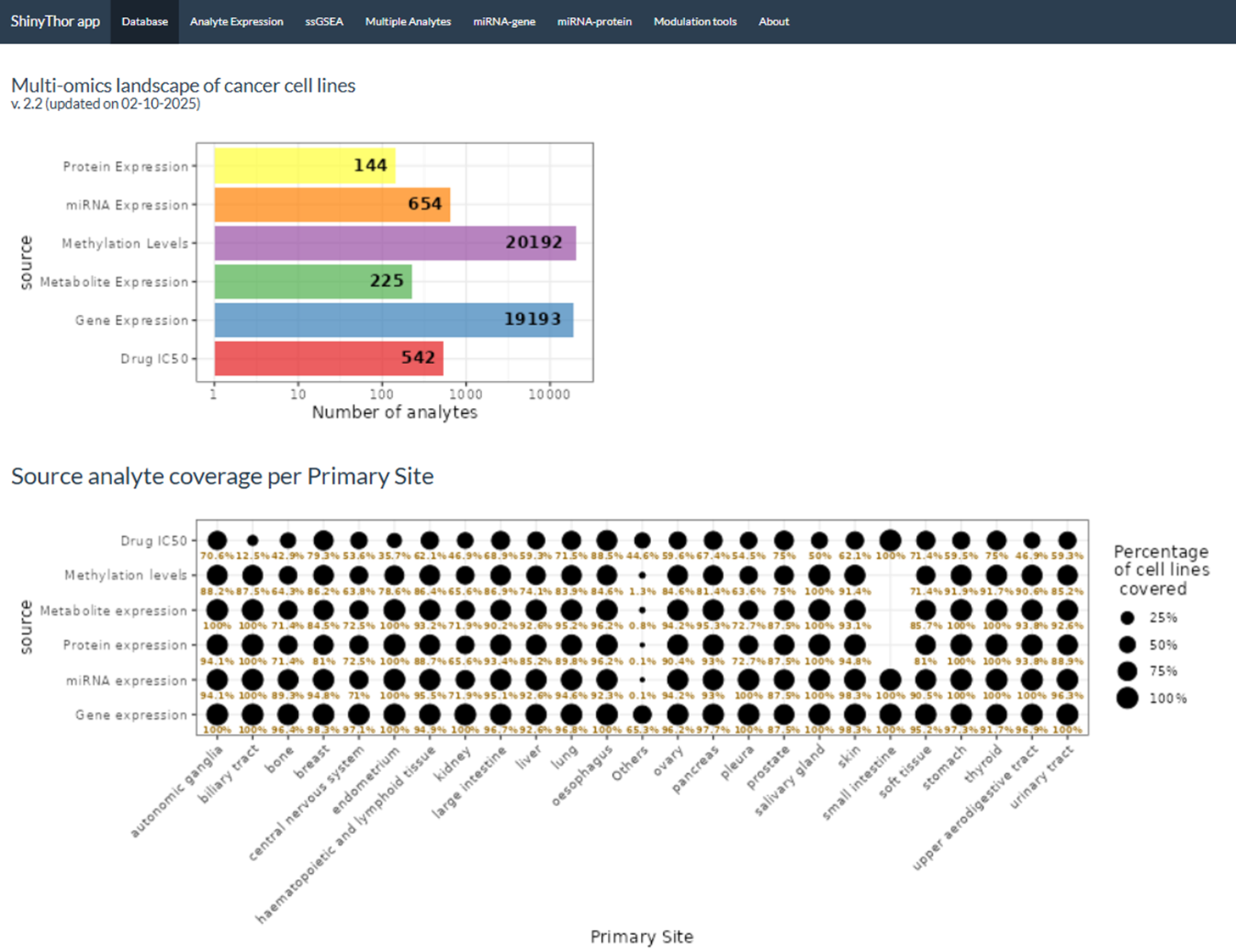


**Figure 1.** Database features. The ShinyThor app includes a cover showing the number of analytes that can be evaluated in the app's current version. At the time of this manuscript, we are on version 2.2 (updated on 02-10-2025). This version includes data from 114 proteins, 654 miRNAs, 20192 methylation levels, 225 metabolites, 19,193 genes, and 542 drugs with IC50 information (top panel). In addition, we can observe the percentage of cell lines mapped for each information (bottom panel). IC50: 50% inhibitory concentration.


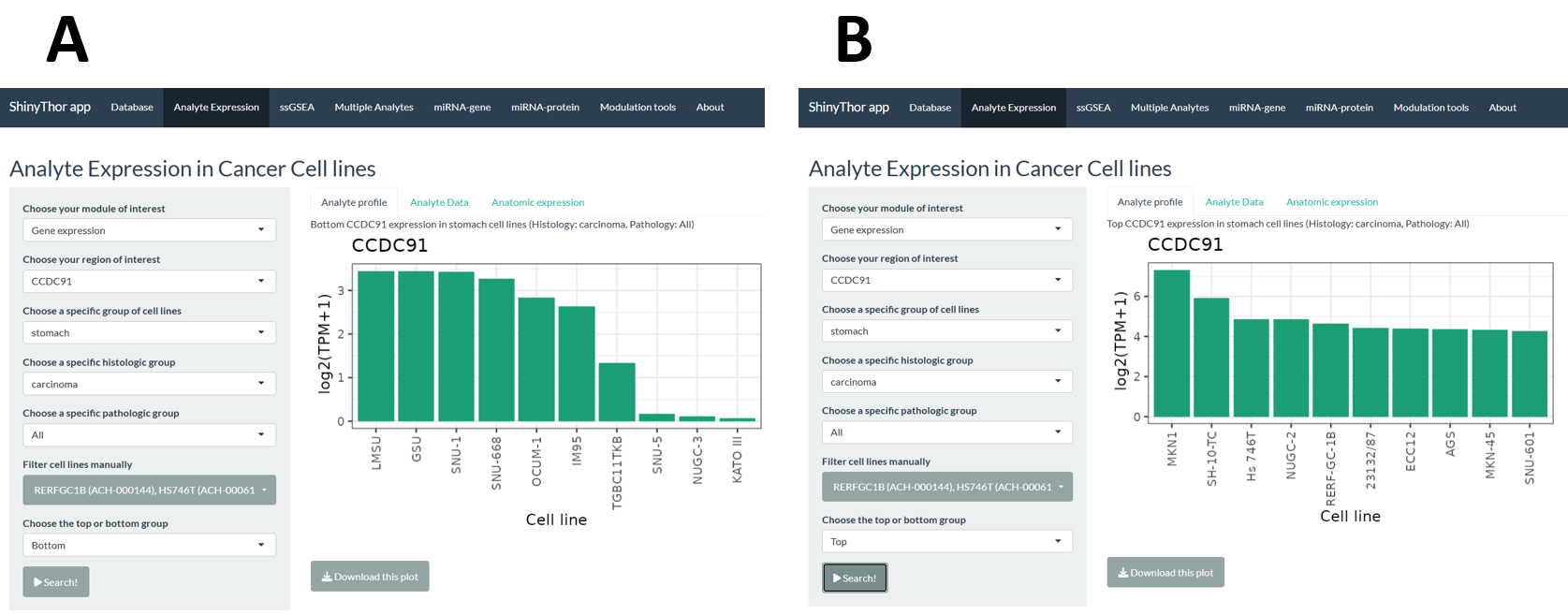


**Figure 2.** Example use of the “Analyte Expression” component. Herein, we can observe the expression profiles for *CCDC91* (part of the GES7 score). Its expression levels are low in KATO III (A), in contrast to AGS (B), where they can be visualized as one of the highest. Note that these images can be downloaded for your use.


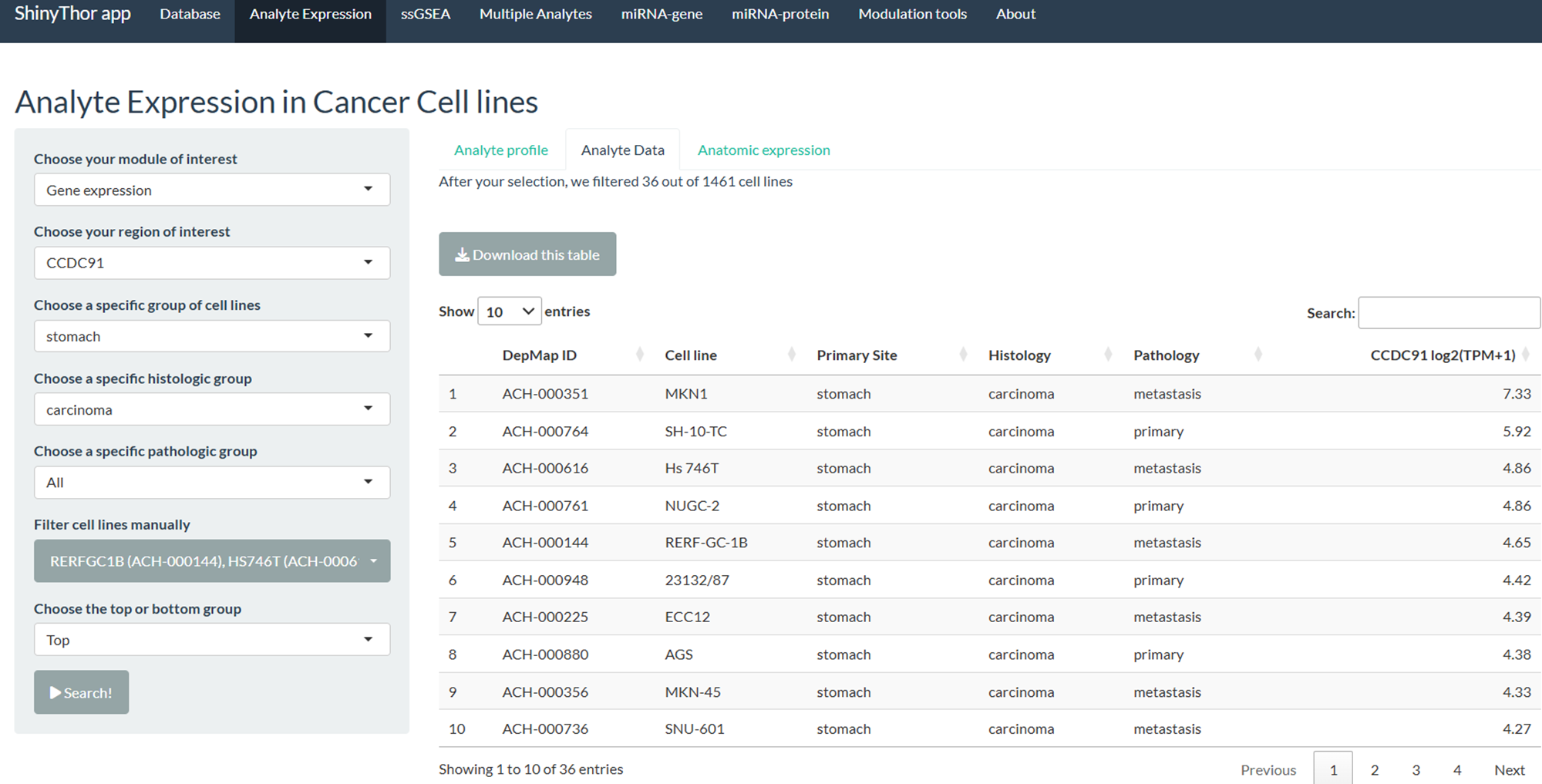


**Figure 3.** Gene expression results can be observed in a table. Following the previous example, the “Analyte Data” tab shows tabular information about the *CCDC91* gene expression levels across all stomach carcinoma cell lines according to their pathologic group.

**
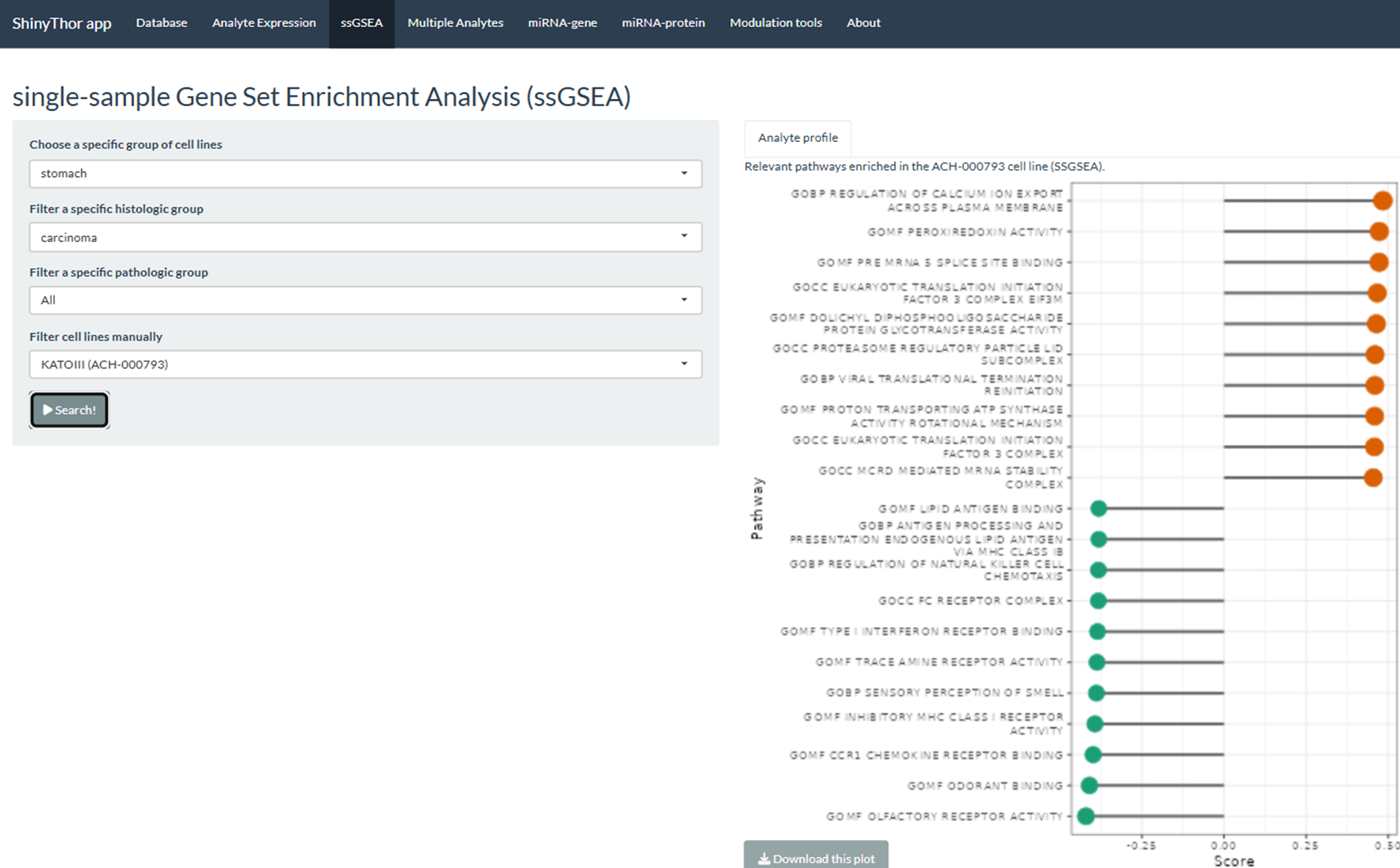
**

**Figure 4.** ssGSEA using cell line transcriptomic levels. Users can perform an enrichment analysis for a specific group of cell lines, histologic and pathologic groups, and finally filter cell lines manually. The plot shows the main enriched and subexpressed pathways in the KATO III cell line according to the calculated ssGSEA score.


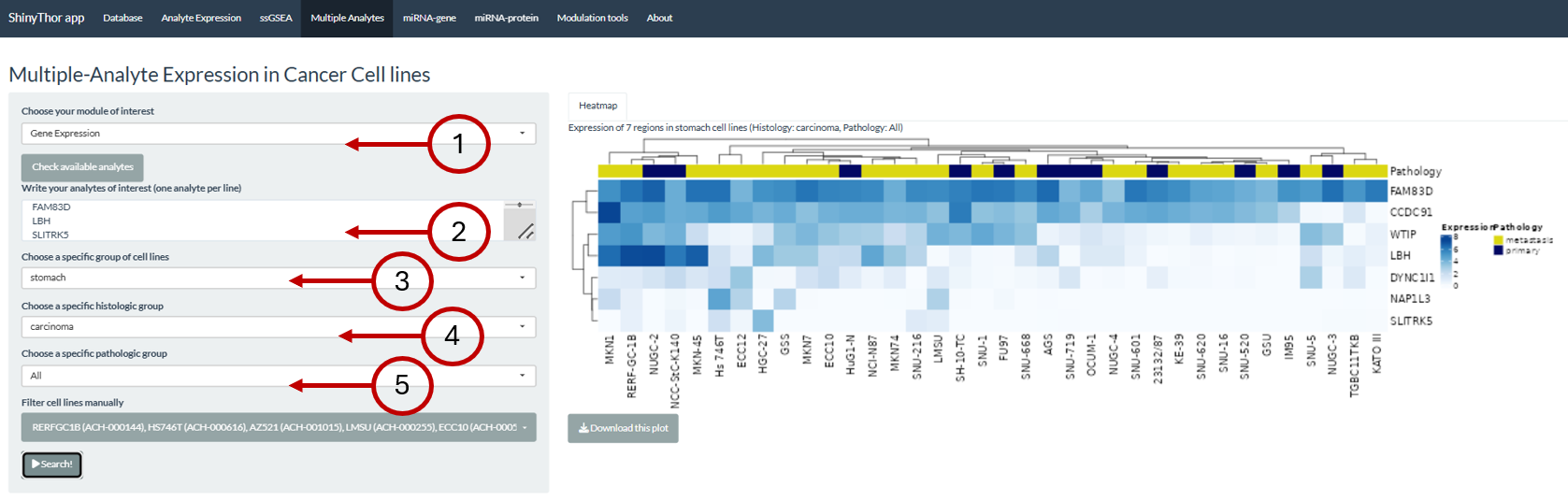


**Figure 5.** Expression profiles for GES7 genes in the *Multiple Analytes* Module. The results are shown in a heatmap in the normalized Expression units and relevant query information. First (1), is necessary to choose a Module (Gene Expression, miRNA Expression, Protein Expression, Metabolite Expression). Second (2), the user might write the regions (genes, proteins, metabolites, or miRNA) to evaluate and visualize the expression levels in a specific group of cell lines (3), histologic (4), and pathologic (5) groups.


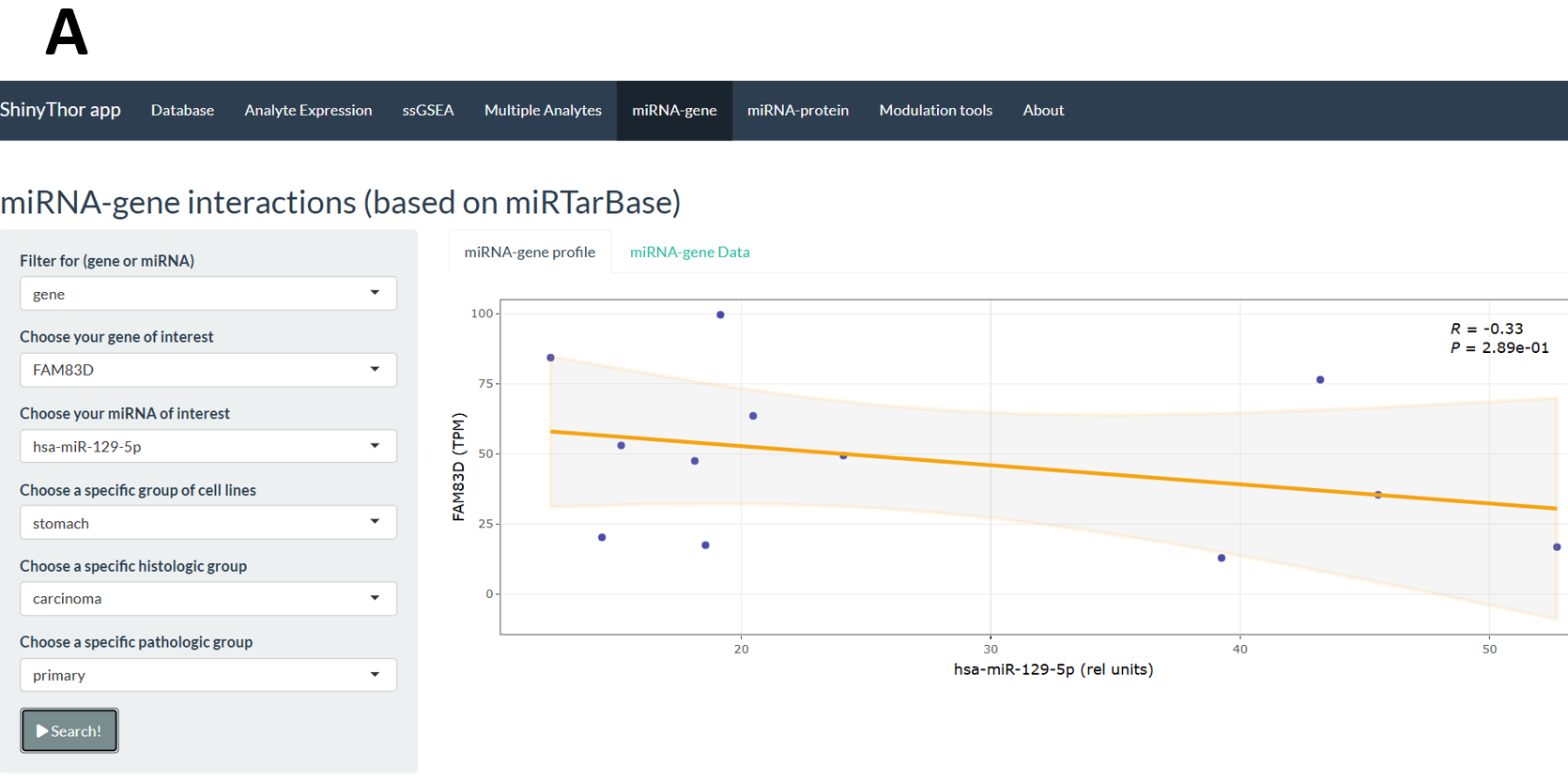


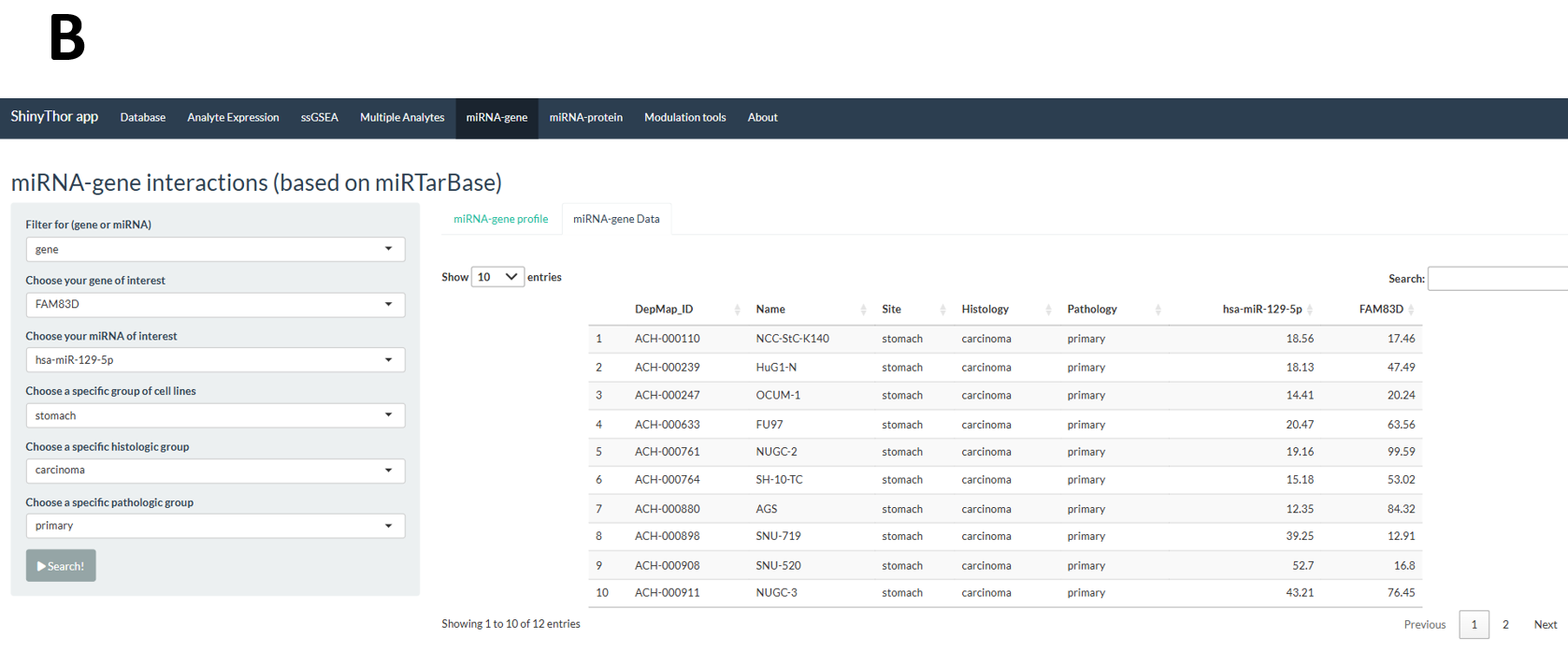


**Figure 6.** Evaluation of the correlation between hsa-miR-129-5p miRNA and *FAM83D* gene in primary stomach cancer cell lines. The plot in panel A shows an XY-plot between the selected miRNA and gene in the CCLE stomach carcinoma cell line in primary lines. Independent of the statistical p-value, it is possible to observe a negative trend between these expression values. Panel B is a table summary of the plot.

**
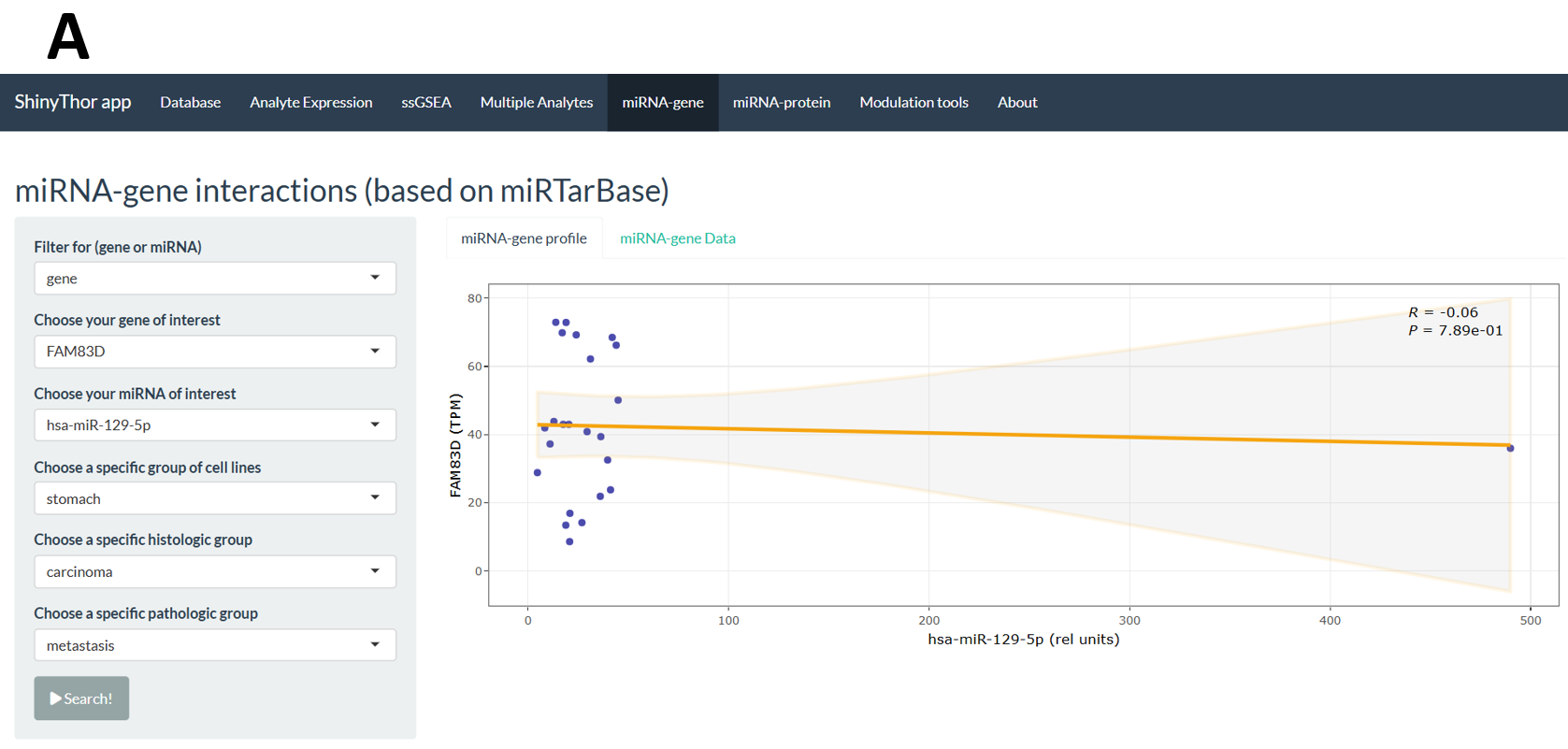
**

**
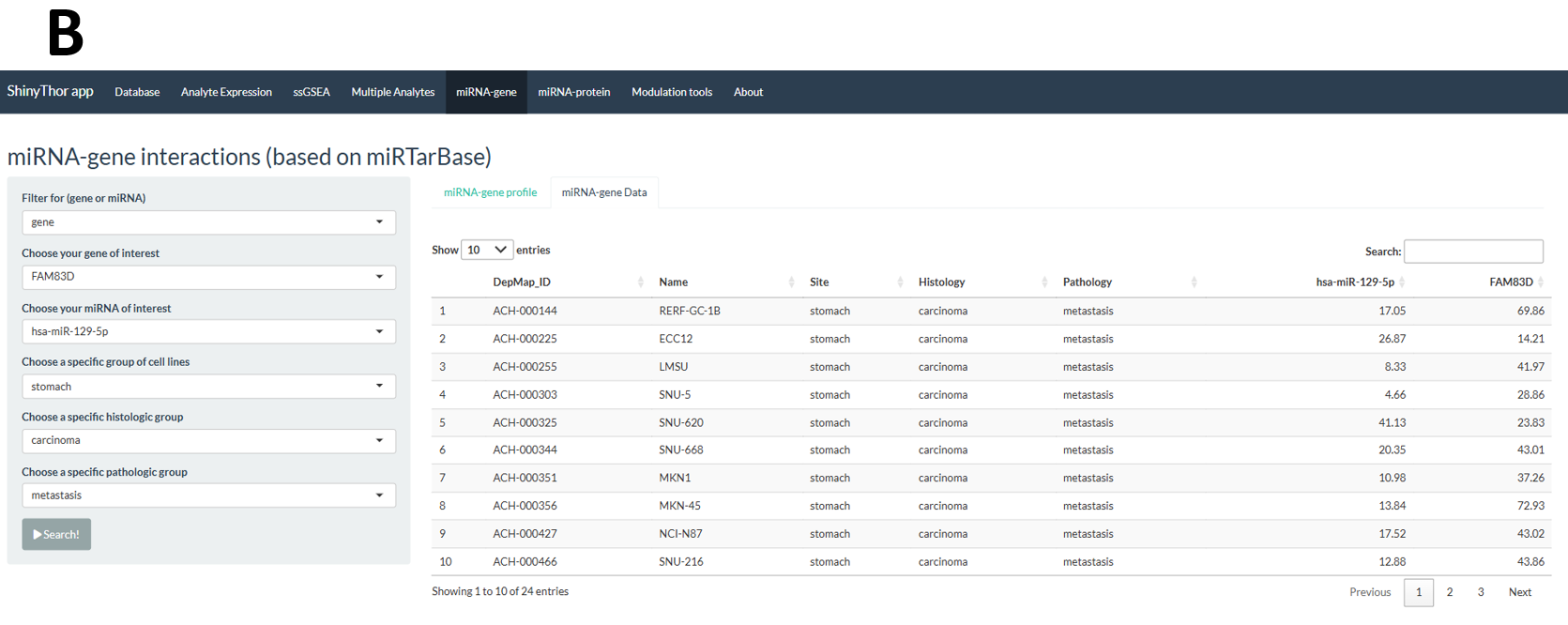
**

**Figure 7.** Evaluation of the correlation between hsa-miR-129-5p miRNA and *FAM83D* gene in metastatic stomach cancer cell lines. Panel A shows the correlation between the same miRNA and gene as in Figure 6. However, in this case, only cell lines derived from metastatic stomach cancer are shown. Note that the negative correlation is not prominent in the plot. Panel B shows a summary of the cell lines and the miRNA-gene values.

**
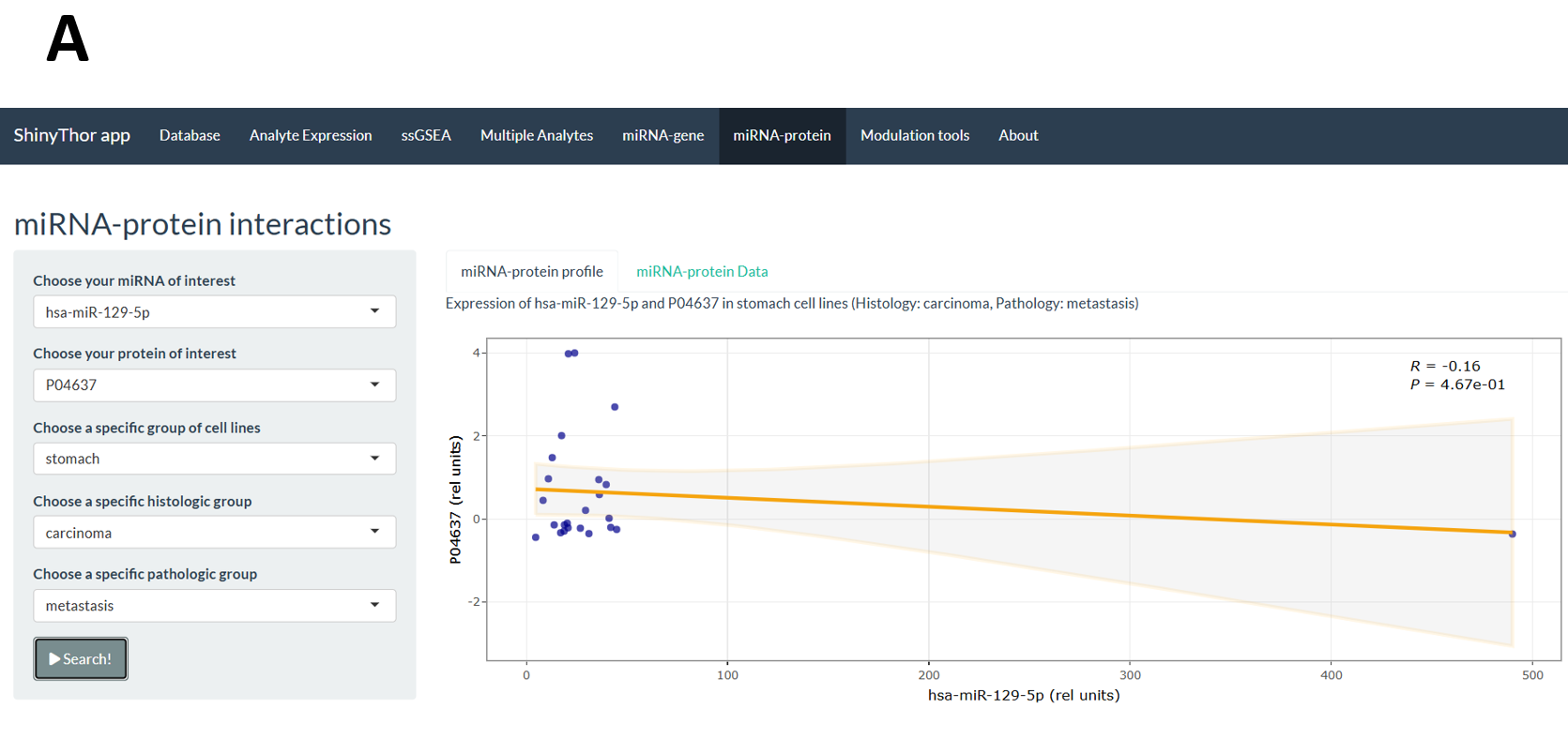
**

**
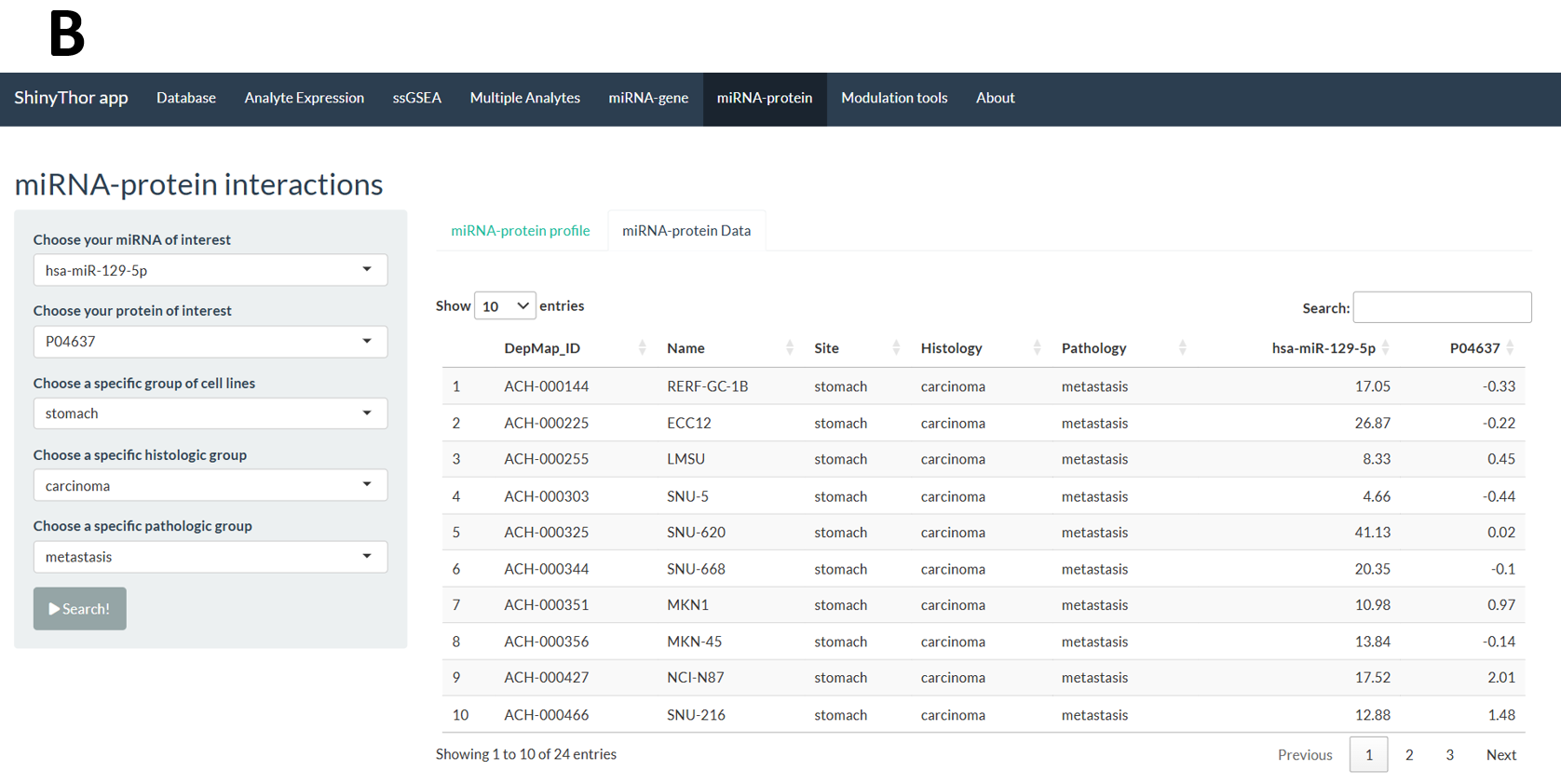
**

**Figure 8.** Evaluation of the correlation between hsa-miR-129-5p miRNA and P04637 protein levels in metastatic stomach cancer cell lines. Panel A shows the correlation between the same miRNA and protein level as in Figure 7. However, in this case, only cell lines derived from metastatic stomach cancer are shown. Note that the negative correlation is not prominent in the plot. Panel B shows a summary of the cell lines and the miRNA-protein levels.

**
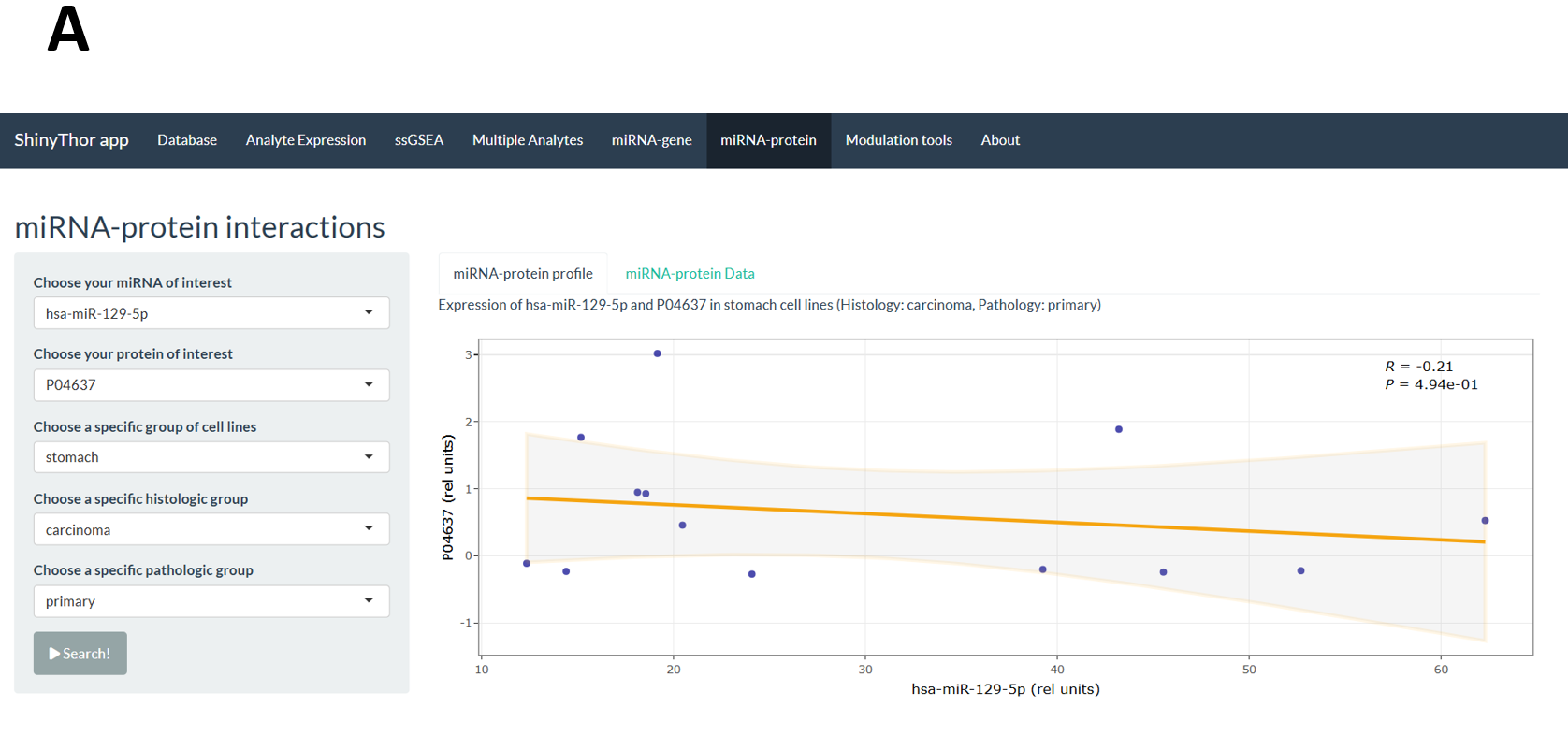
**

**
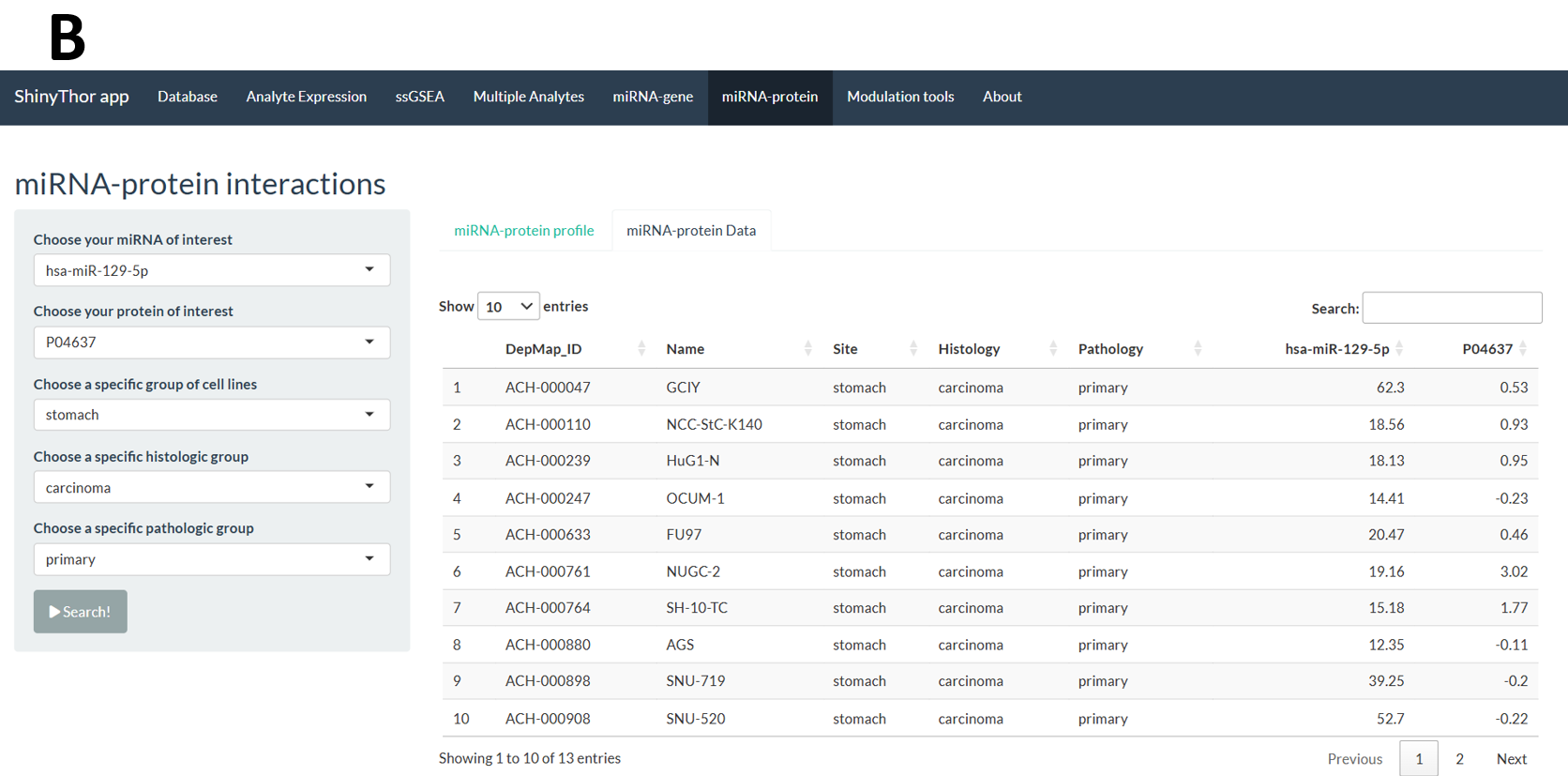
**

**Figure 9.** Similar to **Figure 8**, the plot shows results between the miRNA hsa-mR-129-5p and the protein levels of P04637 in cell lines derived from metastatic stomach cancer (Panel A). Panel B shows the results in table form.


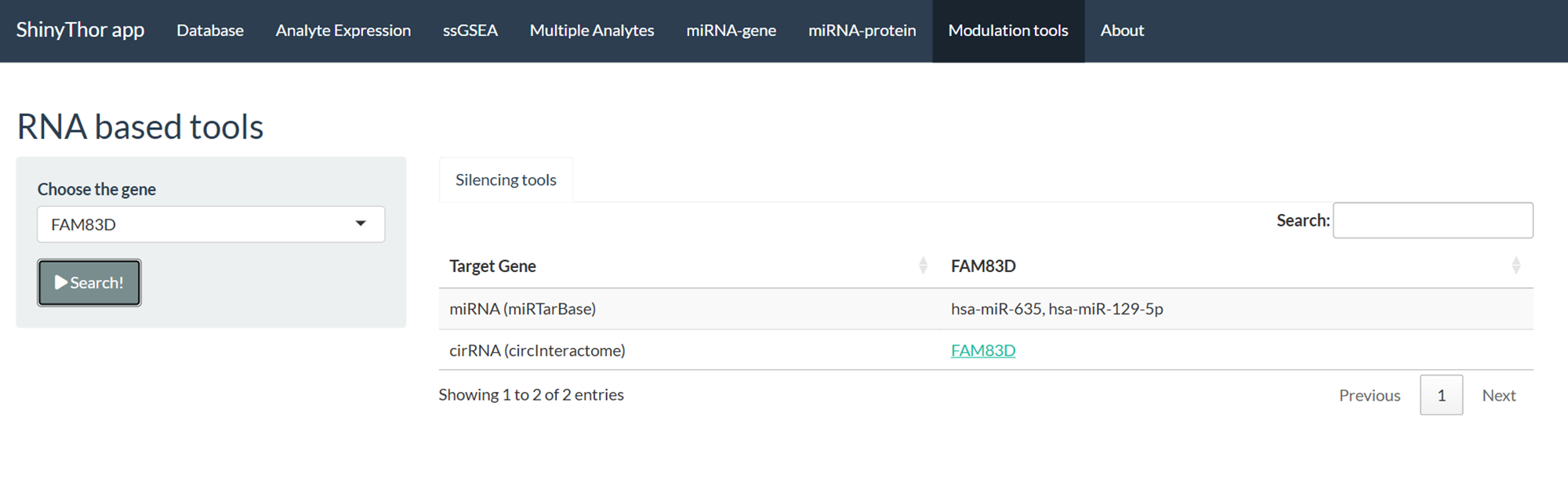


**Figure 10.** Silencing Tools based on miRNA or circRNA for *FAM83D*. After indicating the gene to be silenced, the system shows three results for the *FAM83D* gene: two miRNAs from miRTarBase (hsa-miR-635 and the previously reviewed hsa-miR129-5p) and some cirRNAs that are accessible by clicking on the link to the circInteractome database.
